# Evidence-Based Practices for Advancing Social Connection in Aging Populations

**DOI:** 10.1093/geroni/igaf122.1043

**Published:** 2025-12-31

**Authors:** Jillian Racoosin Kornmeier, Meredith Hanley

**Affiliations:** Foundation for Social Connection, Washington, District of Columbia, United States; USAging, Washington, District of Columbia, United States

## Abstract

This presentation will bring together research and practice experts to examine how factors at the individual, community, and systemic levels contribute to social isolation and loneliness among older adults, shaping both challenges and opportunities for social connection. They will then explore evidence-based and promising strategies for fostering social engagement through the Built Environment, Arts & Culture, and Nutrition sectors. Drawing from the Foundation for Social Connection’s Systems Of Cross-sector Integration and Action across the Lifespan (SOCIAL) Framework reports and their collective expertise, they will highlight how community design, arts-based and cultural programming, and nutrition initiatives can successfully engage older adults. By combining research findings with a discussion of practical, real-world applications, this session will provide attendees with a comprehensive understanding of how these strategies can support older adults in building and sustaining meaningful connections. The session will also examine important considerations for adapting these strategies to diverse community settings and needs. Attendees will leave with a strong grasp of the research behind these approaches and inspiration to implement them in their own communities.

